# The Relationship Between Hippocampal Cerebrovascular Reactivity and Brain Structure in Older Age

**DOI:** 10.1002/hbm.70445

**Published:** 2026-02-18

**Authors:** Congxiyu Wang, Lucy Jobbins, Graham Reid, Georgina Hobden, Raihaan Patel, Clare E. Mackay, Klaus P. Ebmeier, Joana Pinto, Daniel Bulte, Mika Kivimäki, Archana Singh‐Manoux, Sana Suri

**Affiliations:** ^1^ Department of Psychiatry University of Oxford Oxford UK; ^2^ Oxford Centre for Human Brain Activity, Centre for Integrative Neuroimaging, Department of Psychiatry University of Oxford Oxford UK; ^3^ Nuffield Department of Clinical Neurosciences University of Oxford Oxford UK; ^4^ Department of Experimental Psychology University of Oxford Oxford UK; ^5^ Oxford Institute of Clinical Psychology Training and Research University of Oxford Oxford UK; ^6^ Institute of Biomedical Engineering, Department of Engineering Science University of Oxford Oxford UK; ^7^ UCL Brain Sciences University College London London UK; ^8^ Epidemiology of Ageing and Neurodegenerative Diseases, INSERM U1153 (France) Université Paris Cité Paris France

**Keywords:** ageing brain, cerebrovascular reactivity, grey matter volume, hippocampus, white matter microstructure

## Abstract

Cerebral autoregulatory mechanisms such as cerebrovascular reactivity (CVR) are impaired in dementia. However, their associations with brain structure, especially in the hippocampus, remain unclear. We investigated associations between hippocampal CVR and hippocampal volume, white matter microstructural integrity, and white matter hyperintensities in older adults. 163 participants from the Heart and Brain Study received multimodal MRI scans, including T1‐weighted structural imaging, diffusion tensor imaging, fluid‐attenuated inversion recovery imaging at Wave 1 (2012–2014, mean age 68.2, SD 4.4 years) and Wave 2 (2019–2023, mean age 76.9, SD 4.5 years). Participants also received BOLD fMRI scans with a 5% CO_2_ inhalation challenge to measure CVR at Wave 2. Linear regression was used to examine the cross‐sectional associations of hippocampal CVR with brain structure at Wave 2 as well as with changes in brain structure between Waves 1 and 2. Lower hippocampal CVR was associated with lower left hippocampal volume, as well as lower fractional anisotropy, higher mean, radial, and axial diffusivity in the corpus callosum, internal capsule, and fornix at Wave 2. Lower hippocampal CVR was also associated with greater changes in white matter integrity in the corpus callosum, internal capsule, and cingulum bundle between Waves 1 and 2. There were no significant associations between hippocampal CVR and white matter hyperintensities. Our findings highlight hippocampal CVR as a potential imaging marker associated with structural brain changes relevant to cognitive decline. Further longitudinal studies are needed to clarify the directionality of this association.

## Graphical abstract

Lower hippocampal cerebrovascular reactivity was associated with smaller grey matter volume and reduced white matter microstructural integrity in the corpus callosum, internal capsule, and fornix cross‐sectionally, and with longitudinal decline in the corpus callosum, internal capsule, and cingulum. No significant associations were observed with white matter hyperintensities. Our findings highlight hippocampal cerebrovascular reactivity as a potential imaging marker associated with structural brain changes relevant to cognitive decline.

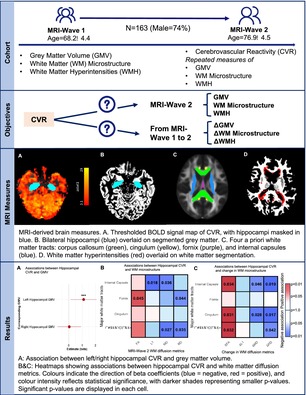

## 1 Introduction

Cognitive decline and related disorders such as dementia are significant challenges for an ageing population. Low cerebral blood flow is an important early physiological change in the dementia pathway (Hachinski et al. 2019), however, the role of autoregulatory mechanisms such as cerebrovascular reactivity (CVR) remains underexplored.

CVR refers to the ability of cerebral blood vessels to dilate in response to vasoactive stimuli and reflects the physiological health of the brain's microvasculature (Liu et al. 2019; Pinto et al. 2021). It complements other more widely studied vascular measures, such as cerebral blood flow and cerebral blood volume, by providing important insights into the vascular reserve of brain tissue (Ryman et al. 2023). Individuals with mild cognitive impairment (MCI), Alzheimer's disease and vascular dementia have lower CVR than cognitively healthy adults (Shim et al. 2015; Cantin et al. 2011; McKetton et al. 2019; Sur et al. 2020), and impairments in CVR may accelerate subsequent cognitive decline (Yezhuvath et al. 2012; Glodzik et al. 2013; Alwatban et al. 2019; Gao et al. 2013). These findings suggest a potential role for CVR in the pathophysiological processes underlying cognitive impairment. However, the association of CVR with neuroimaging biomarkers of dementia, such as grey matter atrophy, white matter degeneration, and cerebrovascular pathology, remains poorly understood. Understanding this relationship could provide insights into the role of vascular health in neurodegeneration.

A key imaging biomarker of dementia is hippocampal atrophy. The hippocampus undergoes a 10%–30% reduction in volume in dementia (Pini et al. 2016), and a smaller left hippocampal volume has been shown to predict conversion from MCI to Alzheimer's disease (Eckerström et al. 2008). Even in cognitively healthy individuals, faster hippocampal atrophy increases vulnerability to cognitive decline (Colliot et al. 2008; Fleischman et al. 2013; Gosche et al. 2002; Jack et al. 2011). Additionally, hippocampal‐associated white matter tracts, such as the cingulum and fornix, undergo demyelination and axonal loss in dementia (Suri et al. 2014). This is typically reflected as changes in white matter (WM) diffusion metrics, such as lower fractional anisotropy (FA) and higher diffusivity indices on diffusion tensor imaging (DTI) scans (Abe et al. 2008; Hakun et al. 2015; Salami et al. 2012; Huang et al. 2012). White matter hyperintensities (WMH), a proxy marker of cerebrovascular lesions, are also linked with cognitive deficits (Van Der Flier et al. 2005; Frisoni et al. 2007), Alzheimer's disease (Garnier‐Crussard et al. 2023; Wang et al. 2020), and vascular dementia (Gootjes et al. 2004). However, the associations between CVR and these structural biomarkers remain unclear and warrant investigation.

This study examined the association between hippocampal CVR and brain structure in the Heart and Brain Study, a cohort of older adults (> 65 years old) who received multi‐modal structural MRI scans at baseline (MRI‐Wave 1) and follow‐up (MRI‐Wave 2, ~9 years later), as well as CVR scans at MRI‐Wave 2 (Suri et al. 2021). CVR was measured non‐invasively using CO_2_ inhalation during a blood oxygen level–dependent (BOLD) magnetic resonance imaging (MRI) scan (Liu et al. 2019; Sleight et al. 2021; Chen 2018). The CO_2_‐based CVR measurement effectively acts as a “stress test” for cerebral circulation and can indicate early signs of vascular dysfunction (Glodzik et al. 2013). We examined three brain structural biomarkers: hippocampal volume, white matter (WM) microstructural integrity, and WMH. For WM microstructure, we selected four white matter tracts a priori (fornix, cingulum, internal capsule, and corpus callosum) that are either associated with the hippocampus or have established roles in higher‐order cognitive functions and are known to be impaired in dementia (Suri et al. 2014). We hypothesised that lower hippocampal CVR at MRI‐Wave 2 would be associated with (i) poorer cross‐sectional measures of brain structure at MRI‐Wave 2, and (ii) greater longitudinal brain structural decline during the preceding 9 years (between MRI‐Waves 1 and 2).

## 2 Material and Methods

### 2.1 Cohort

The Heart and Brain Study (HBS; conducted in 2019–2023) includes 163 participants selected from 775 individuals who took part in the Whitehall II (WHII) Imaging study (conducted in 2012–2016). The WHII Imaging study is a sub‐study of the WHII cohort of British civil servants, established in 1985 (Marmot and Brunner 2005). The participants underwent baseline 3 T brain MRI scans at the mean age of 68.2 ± 4.4 years as part of the WHII Imaging Study (referred to as MRI‐Wave 1 (Filippini et al. 2014)) and again at the mean age of 76.9 ± 4.5 years for HBS (referred to as MRI‐Wave 2 (Suri et al. 2021)) at the Oxford University Centre for Integrative Neuroimaging (detailed protocols are available in (Suri et al. 2021; Filippini et al. 2014)).

Eligibility criteria for this study included no self‐reported clinical diagnoses of dementia at MRI‐Waves 1 and 2, no contraindications for MRI (e.g., pacemaker or claustrophobia) or the CVR scan (e.g., severe asthma, chronic obstructive pulmonary disease), and no significant incidental findings on the MRI scans (e.g., stroke, tumour). All participants provided written informed consent during their visit. This study received ethical approval from the Medical Sciences Interdivisional Research Ethics Committee of the University of Oxford, under the reference: R57135/RE006. The study was conducted in accordance with the University of Oxford's Research Ethics Policy, which is consulted in conjunction with the University's Code of Practice and Procedure on Academic Integrity in Research and reflects the principles and commitments outlined in the funder‐endorsed Concordat to Support Research Integrity (UKCORI 2015).

### 2.2 MRI Acquisitions and Imaging Data Pre‐Processing

Participants received 3T T1‐weighted structural MRI, DTI, Fluid‐Attenuated Inversion Recovery (FLAIR) at MRI‐Waves 1 and 2, as well as a CO_2_‐CVR scan only at MRI‐Wave 2. Scan protocols were closely matched between waves; however, they were conducted on different scanners (MRI‐Wave 1: 3T Verio; MRI‐Wave 2: 3T Prisma). Detailed sequence parameters for these scans are provided in the HBS protocol paper (Suri et al. 2021), as well as in Supplementary Table 1. Imaging processing was performed using FMRIB Software Library (FSL) tools (Jenkinson et al. 2012). Details of the pre‐processing of GMV, CVR, WM microstructure and WMH scans are described in the HBS protocol paper (Suri et al. 2021) and presented briefly here.

### 2.3 Grey Matter Volume (GMV)

For both waves, GMV was extracted from T1‐weighted MRI scans using the automated FSL_ANAT pipeline, which performs gradient distortion correction, bias correction, brain extraction, and registration to the MNI152 standard space (Jenkinson et al. 2012). Brain tissue segmentation into grey matter, white matter, and cerebrospinal fluid was carried out using FSL_FAST (Zhang et al. 2001), which generates partial volume estimates to minimise tissue boundary contamination. Segmentation of the bilateral hippocampus was performed using FSL_FIRST on the partial volume estimates, and the final segmentations were visually inspected by CW for accuracy (Patenaude et al. 2011). The total grey matter and bilateral hippocampal volumes were extracted and converted to cm^3^ for statistical analysis.

### 2.4 Cerebrovascular Reactivity (CVR)

CVR was assessed using a hypercapnia challenge during a BOLD fMRI scan at MRI‐Wave 2 only. Participants were fitted with an anaesthetic face mask through which they inhaled controlled levels of gases following a structured paradigm: an initial 60‐s of medical air, followed by two 75‐s blocks of 5% CO_2_ in medical air, interleaved with 75‐s blocks of medical air. This paradigm was extensively piloted and optimised (Suri et al. 2021) and has been shown to produce cerebrovascular responses comparable to longer continuous CO_2_ challenges while allowing for minimal participant discomfort, particularly suitable for the older cohort in this study (Liu et al. 2018; Yezhuvath et al. 2009). The end‐tidal CO_2_ (EtCO_2_, the maximum CO_2_ concentration at the end of an exhaled breath) was recorded throughout the scan and extracted using a customised MATLAB script (Suri et al. 2021; Bulte et al. 2012). The EtCO_2_ capnometry trace was visually inspected for each participant to ascertain that the EtCO_2_ reached a steady state within each CO_2_ challenge block (Supporting Information Figure 1), and the baseline EtCO_2_ after each block remained comparable to the initial baseline (Suri et al. 2021). For each participant, the normalised EtCO_2_ was calculated (by subtracting the baseline mean and dividing by the maximum EtCO_2_) and then was used as a regressor to correlate with the BOLD signal time‐course using FSL_FEAT's general linear model (Woolrich et al. 2001). CVR was quantified as the percentage change in BOLD signal per unit change in raw EtCO_2_ (%BOLD/ΔEtCO_2_ mmHg), where ΔEtCO_2_ represents the difference between the average EtCO_2_ levels achieved during hypercapnia (calculated using the second half of each hypercapnia block where ETCO_2_ levels were stabilised) and normocapnia (computed using the initial normocapnic EtCO_2_ period). Mean CVR was extracted from regions of interest (ROIs) which included the total grey matter and the left and right hippocampus using Featquery. Hippocampal masks were derived from each participant's T1‐weighted hippocampal segmentation and then registered to CVR space to ensure alignment between the regions used for GMV and CVR extraction.

### 2.5 White Matter (WM) Microstructure

**FIGURE 1:**
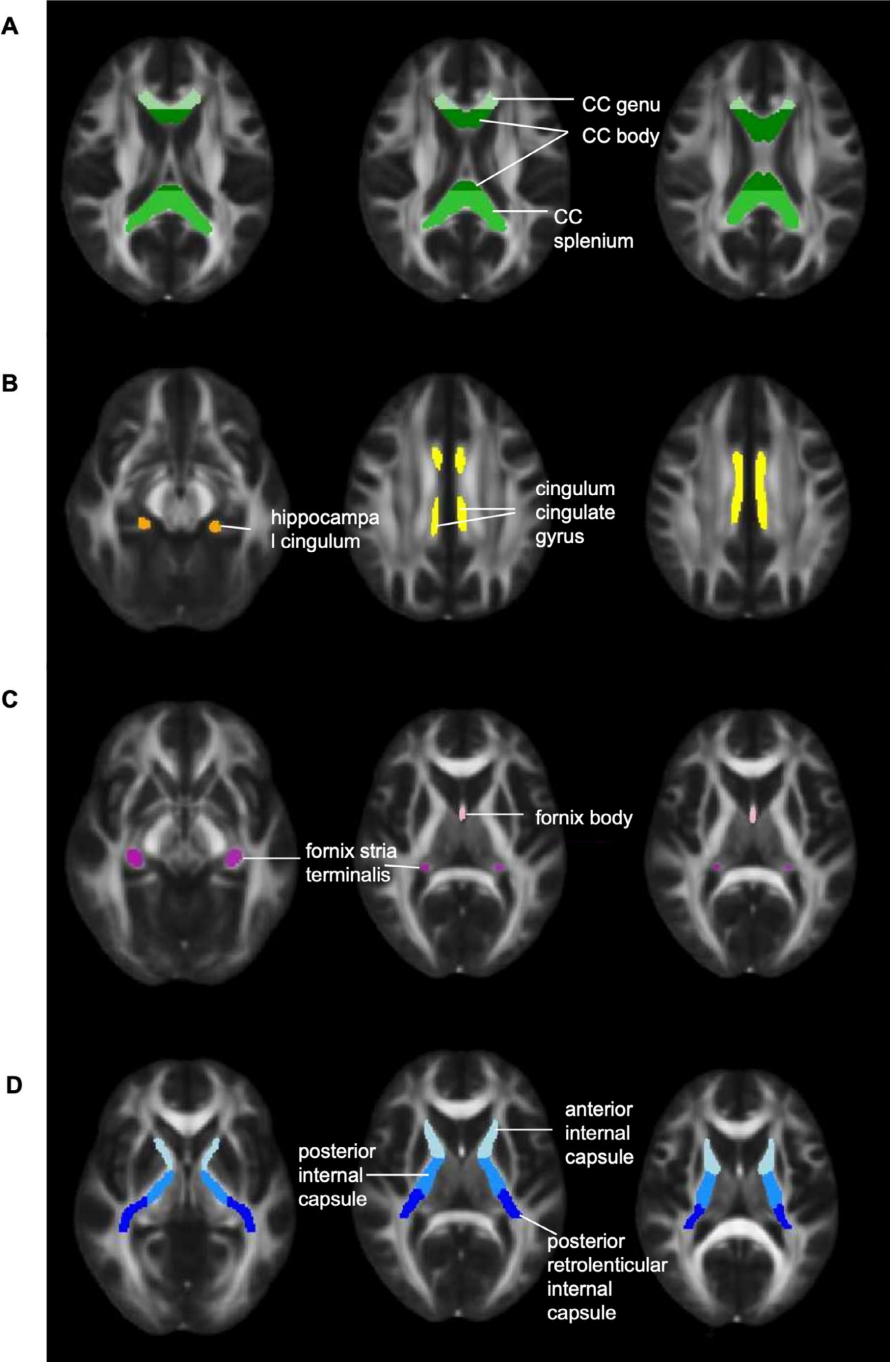
Regions of interest for four major white matter tracts. Masks are overlaid FMRIB58_FA image. (A) Corpus Callosum: Body (dark green), splenium (green), genu (light green). (B) Cingulum Bundle: Bilateral hippocampal cingulum (orange), bilateral cingulate gyrus (yellow). (C) Fornix: Bilateral fornix stria terminalis (purple), fornix body (pink). (D) Internal Capsule: Bilateral anterior (light blue), bilateral posterior (blue), bilateral posterior retrolenticular (dark blue)

At both waves, WM microstructural integrity was assessed by the diffusion metrics derived from DTI scans. These scans were processed with the FMRIB Diffusion Toolbox which included FSL_TOPUP, FSL_EDDY and dtifit to correct for susceptibility‐induced distortions, eddy current and motion artefacts, and to generate voxel‐wise diffusivity maps, respectively (Smith et al. 2006; Andersson and Sotiropoulos 2016). These maps were entered into Tract‐Based Spatial Statistics (TBSS) (Smith et al. 2006) to compute fractional anisotropy (FA), mean diffusivity (MD), radial diffusivity (RD), and axial diffusivity (L1), where higher FA and lower diffusivity generally indicate better WM microstructural integrity (Alexander et al. 2007). ROI masks were derived from the Johns Hopkins University (JHU) ICBM‐DTI 81 white matter atlas (Mori et al. 2008), encompassing 25 tracts for each hemisphere. We selected four principal white matter tracts that are related to cognitive decline in dementia: the corpus callosum, fornix, cingulum bundle, and internal capsule (Suri et al. 2014; Jin Thong et al. 2014). For each tract, masks of individual segments were combined into a single tract mask (e.g., body, genu and splenium of corpus callosum were combined to form a corpus callosum tract; Figure 1). The combined mask was then used to extract the values of FA, MD, RD, and L1 from the TBSS‐derived skeleton for each participant at each time point.

### 2.6 White Matter Hyperintensities Volume (WMH)

WMH were quantified from T2‐weighted FLAIR scans at both waves. WMH probability maps were generated by FSL_BIANCA and thresholded at 0.9 (voxels with greater than 90% probability of being WMH) to extract the total WMH volume (Griffanti et al. 2016). This was then expressed as a percentage of total brain volume (sum of grey matter, white matter, and cerebrospinal fluid; expressed as %WMH) and log‐transformed to reduce skewness and normalise the distribution of %WMH at both waves, as has been done before in this cohort (Hobden et al. 2025).

### 2.7 Longitudinal Change in Brain Structure

To account for inter‐scanner variability between waves 1 and 2, we standardised (z‐scored) the GMV and WM diffusion metrics values within each wave using the R “scale” function to ensure that the measures were comparable between the waves. Longitudinal changes in GMV/WM diffusion metrics were calculated as the difference between standardised values from MRI‐Wave 2 and MRI‐Wave 1 (e.g., ΔGMV = standardised GMV at MRI‐Wave 2—standardised GMV at MRI‐Wave 1; ΔFA = standardised FA at MRI‐Wave 2—standardised FA at MRI‐Wave 1 and similarly for MD, RD, L1). Since we are using standardised values, the changes do not reflect absolute change at the individual level but rather the relative amount of change for an individual compared to others within the cohort. This approach enables assessment of relative change (i.e., whether a person's hippocampus underwent more/less change relative to other participants) but does not allow inferences about absolute volumetric change (i.e., whether the volume of a person's hippocampus increased/decreased from MRI‐Wave 1 to 2). It is reasonable to assume, based on extensive literature, that in the age range of 70–80 years, hippocampal volume and FA within our selected tracts are more likely to decrease than increase (Damoiseaux et al. 2009; Barnes et al. 2009), while diffusivity (MD, RD, L1) is more likely to increase (Madden et al. 2012; Sullivan and Pfefferbaum 2006). Thus, for GMV and FA, we can reasonably conclude that ***more negative*** standardised Δ values reflect more change (i.e., more atrophy and reduced WM microstructural integrity) relative to the rest of the group. In contrast, for MD, RD, and L1, ***more positive*** standardised Δ values reflect more change (i.e., reduced WM microstructural integrity) relative to the rest of the group (see Supporting Information Figure 2 for distributions of Δ values across all dependent variable measures).

We did not standardise WMH values at each MRI wave because we have previously shown that the performance of the FSL_BIANCA tool is consistent over the two scanners (as described in the cohort protocol paper (Suri et al. 2021)). Longitudinal change in WMH volumes between waves was therefore calculated as the absolute difference in log‐transformed %WMH values (ΔWMH = MRI‐Wave 2 logWMH%—MRI‐Wave 1 logWMH%), that is, positive values represent an increase in WMH volumes from MRI‐Wave 1 to 2.

### 2.8 Statistical Analysis

Statistical analyses were conducted using RStudio (version 4.3.0). Associations between CVR and dependent variables were examined using linear regression. The Shapiro–Wilk test was used to assess the model residuals and ensure normality for linear regression models (Shapiro and Wilk 1965).

We examined the cross‐sectional association of hippocampal CVR with (1) hippocampal GMV, (2) FA, MD, RD and L1 of four predefined white matter tracts, and (3) WMH levels at MRI‐Wave 2, adjusting for sex and age. For hippocampal GMV analysis, we examined the left and right hippocampus separately. In the WM microstructure and WMH analysis, hippocampal CVR was defined as the average of the left and right hippocampal CVR values.

We also examined how hippocampal CVR at MRI‐Wave 2 was associated with longitudinal changes over the preceding 9 years in the following MRI measures: (1) ΔGMV of hippocampus, (2) ΔWM diffusion metrics, and (3) ΔWMH between MRI‐Wave 1 and 2. Age at MRI‐Wave 1, time between waves, and sex were included as covariates in the longitudinal models.

Additionally, in a supplementary analysis, we examined the associations of whole‐brain CVR with the same MRI measures as well as with whole‐brain values of GMV, FA, MD, RD, and L1 in order to assess whether our observed associations were specific to hippocampal CVR or whether they were globally diffuse.

Associations with *p* < 0.05 were considered statistically significant and reported in the results. We additionally corrected for multiple comparisons across the four WM tracts using the Benjamini‐Hochberg (BH) correction (Benjamini and Hochberg 1995) and report p_corr_ < 0.05 for the WM analyses. The BH method uses a ranking‐based approach to control the false discovery rate (FDR) and is widely used in neuroimaging research (Genovese et al. 2002; Sun et al. 2025; Clark et al. 2020). Cohen's f^2^ values for the whole regression model and for each independent variable were used to assess effect sizes for significant associations.

## 3 Results

### 3.1 Participant Characteristics

Of the 163 participants at MRI‐Wave 2, 74% were male, approximately reflecting the distribution of the British civil service at cohort inception. Eight participants were excluded due to factors such as movement or discomfort in the MRI scanner or incompatibility with the CVR mask. A further single CVR scan was excluded as an outlier (|CVR—median| > 6 × Median absolute deviation), resulting in 154 participants for the final analysis (female/male = 40/114, 26%/74%). Mean age at MRI‐Wave 1 was 68.2, SD = 4.4 years and mean age at MRI‐Wave 2 was 76.9, SD = 4.5 years. The mean ± SD time between waves was 8.7 ± 1.2 years. Hippocampal CVR was negatively associated with age at MRI‐Wave 2 (left: *β* = −0.003, *p* = 0.005; right: *β* = −0.004, *p* = 0.005). Descriptions of the MRI markers at MRI‐Wave 2 are summarised in Table 1.

**TABLE 1:** MRI markers of participants at MRI‐Wave 2 (*N* = 154 with complete MRI).

		Mean (SD)
Whole Brain CVR (%/mmHg)		0.28 (0.07)
Left Hippocampal CVR (%/mmHg)		0.21 (0.07)
Right Hippocampal CVR (%/mmHg)		0.20 (0.07)
Whole Brain GMV (cm^3^)		531.69 (45.39)
Left Hippocampal GMV (cm^3^)		2.92 (0.44)
Right Hippocampal GMV (cm^3^)		3.07 (0.40)
FA	Whole Brain	0.47 (0.02)
	Corpus Callosum	0.72 (0.03)
	Cingulum Bundle	0.60 (0.03)
	Internal Capsule	0.64 (0.02)
	Fornix	0.50 (0.04)
MD (mm^2^/s)	Whole Brain	6.98 × 10^−4^ (2.95 × 10^−5^)
	Corpus Callosum	6.94 × 10^−4^ (4.18 × 10^−5^)
	Cingulum Bundle	6.56 × 10^−4^ (2.93 × 10^−5^)
	Internal Capsule	6.28 × 10^−4^ (2.59 × 10^−5^)
	Fornix	8.47 × 10^−4^ (6.62 × 10^−5^)
RD (mm^2^/s)	Whole Brain	5.01 × 10^−4^ (3.16 × 10^−5^)
	Corpus Callosum	3.11 × 10^−4^ (4.51 × 10^−5^)
	Cingulum Bundle	3.85 × 10^−4^ (3.31 × 10^−5^)
	Internal Capsule	3.45 × 10^−4^ (2.79 × 10^−5^)
	Fornix	6.03 × 10^−4^ (8.38 × 10^−5^)
L1 (mm^2^/s)	Whole Brain	1.09 × 10^−3^ (2.79 × 10^−5^)
	Corpus Callosum	1.46 × 10^−3^ (4.81 × 10^−5^)
	Cingulum Bundle	1.20 × 10^−3^ (4.17 × 10^−5^)
	Internal Capsule	1.19 × 10^−3^ (3.68 × 10^−5^)
	Fornix	1.33 × 10^−3^ (4.99 × 10^−5^)
White Matter Hyperintensity (logWMH%)		1.55 (0.49)

*Note:* GMV, FA, MD, RD, L1 and WMH markers were also acquired at Wave 1. However, these values were standardised as described in the Materials and Methods and therefore not presented below.

Abbreviations: CVR = cerebrovascular reactivity, FA = fractional anisotropy, GMV = grey matter volume, L1 = axial diffusivity, Max = maximum, MD = mean diffusivity, Min = minimum, SD = standard deviation.

### 3.2 Associations Between Hippocampal CVR and GMV

**FIGURE 2:**
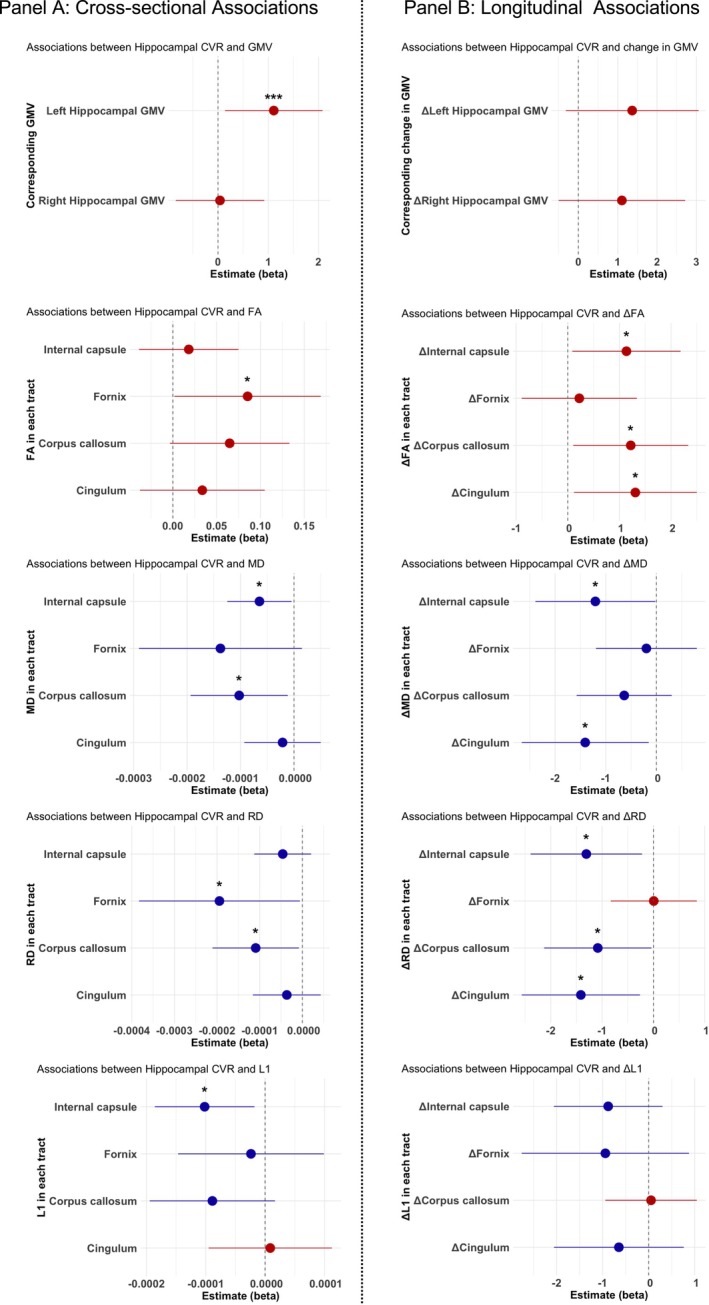
Forest plots showing associations of hippocampal CVR with GMV and diffusion metrics (FA, MD, RD, and L1) across the four white matter tracts. Panel A shows cross‐sectional associations adjusted for age and sex. Panel B shows longitudinal (Δ) associations adjusted for age at MRI‐Wave 1, sex, and time interval between MRI‐Wave 1 and 2. Lines represent the 95% confidence intervals. Asterisks indicate statistical significance (*p < 0.05; ***p < 0.001). Red indicates positive associations; blue indicates negative associations.

**FIGURE 3:**
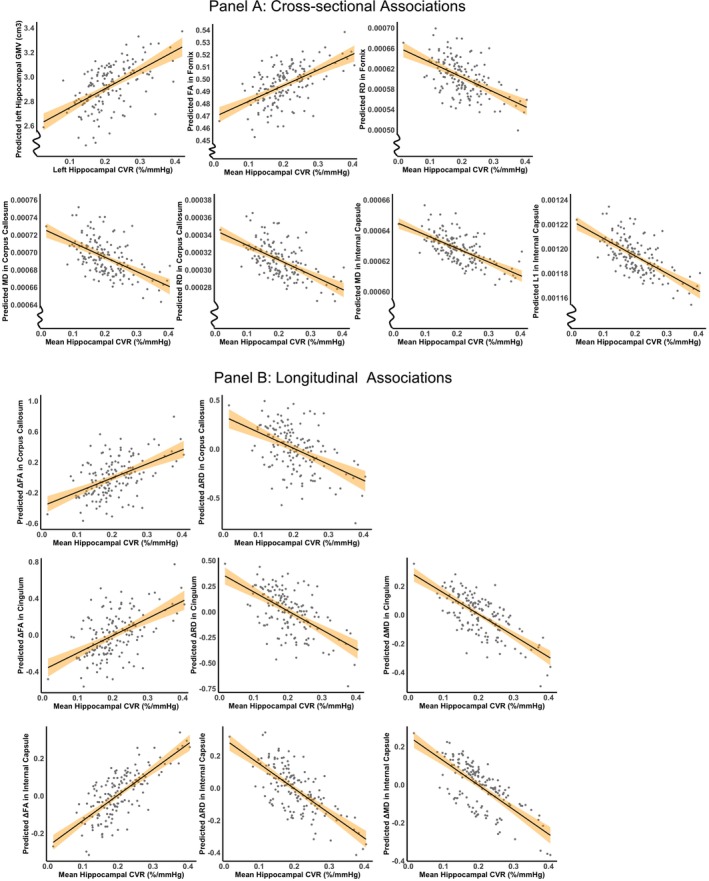
Scatter plots of significant associations between hippocampal CVR and brain structure. The *y*‐axis represents predicted values from linear regression models. Panel A shows cross‐sectional associations adjusted for age and sex. Panel B shows longitudinal associations adjusted for age at MRI‐Wave 1, sex, and time interval between MRI‐Waves 1 and 2. Shaded areas represent the 95% confidence intervals of the regression estimates. ΔFA, ΔMD, ΔRD, and ΔL1 represent changes in the standardised values of these metrics between waves. A larger negative ΔFA indicates a greater change (i.e., reduction) in FA for an individual relative to others in the cohort. Conversely, larger positive ΔMD, ΔRD, or ΔL1 values suggest greater increases in these metrics for an individual than for the cohort.

Lower left hippocampal CVR was significantly associated with lower left hippocampal volume at MRI‐Wave 2 (*β* = 1.11, *p* = 0.025) after adjusting for age, sex and total GMV (Table 2, Figures 2A and 3A). However, no significant associations were observed between CVR and volume in the right hippocampus (*β* = 0.04, *p* = 0.928).

**TABLE 2:** Summary of statistical associations between hippocampal CVR and structural measures at MRI‐Wave 2.

	Independent variable	Dependent variable	*β*	95% CI	*p*	pcorr	Cohen's f^2^ Overall model (Independent variable)
GMV	Left hippocampus CVR	Left hippocampus GMV	1.11	[0.14, 2.08]	0.025*	—	0.22 (0.03)
	Right hippocampus CVR	Right hippocampus GMV	0.04	[−0.84, 0.92]	0.928	—	—
WM microstructure	FA						
	Hippocampal CVR	Corpus callosum	0.06	[−0.003, 0.13]	0.063	0.125	—
		Cingulum bundle	0.03	[−0.04, 0.10]	0.353	0.471	—
		Internal capsule	0.02	[−0.04, 0.07]	0.529	0.529	—
		Fornix	0.09	[0.002, 0.17]	0.045*	0.125	0.21 (0.03)
	MD						
	Hippocampal CVR	Corpus callosum	−1.0 × 10^−4^	[−1.9 × 10^−4^, −1.2 × 10^−5^]	0.027*	0.071	0.32 (0.03)
		Cingulum bundle	−2.1 × 10^−5^	[−9.3 × 10^−5^, 5.0 × 10^−5^]	0.556	0.556	—
		Internal capsule	−6.5 × 10^−5^	[−1.3 × 10^−4^, −4.4 × 10^−6^]	0.036*	0.071	0.15 (0.02)
		Fornix	−1.4 × 10^−4^	[−2.9 × 10^−4^, 1.5 × 10^−5^]	0.077	0.102	—
	RD						
	Hippocampal CVR	Corpus callosum	−1.1 × 10^−4^	[−2.1 × 10^−4^, −8.1 × 10^−5^]	0.035*	0.087	0.23 (0.03)
		Cingulum bundle	−3.6 × 10^−5^	[−1.2 × 10^−4^, 4.3 × 10^−5^]	0.368	0.368	—
		Internal capsule	−4.6 × 10^−5^	[−1.1 × 10^−4^, 2.1 × 10^−5^]	0.175	0.233	—
		Fornix	−1.9 × 10^−4^	[3.8 × 10^−4^, −5.7 × 10^−6^]	0.044*	0.087	0.23 (0.03)
	L1						
	Hippocampal CVR	Corpus callosum	−8.9 × 10^−5^	[−1.9 × 10^−4^, −1.7 × 10^−5^]	0.098	0.197	—
		Cingulum bundle	−8.7 × 10^−6^	[−9.5 × 10^−5^, 1.1 × 10^−4^]	0.869	0.869	—
		Internal capsule	−1.0 × 10^−4^	[−1.9 × 10^−4^, −1.8 × 10^−5^]	0.018*	0.071	0.20 (0.04)
		Fornix	−2.4 × 10^−5^	[−1.5 × 10^−4^, 9.9 × 10^−5^]	0.702	0.869	—
WMH	Hippocampal CVR	LogWMH%	−0.19	[−1.37, 1.00]	0.756	—	—

Abbreviations: β = coefficient of the linear regression models, Cohen's f^2^ to indicate effect size for significant results, (table displays both Cohen's f^2^ for the overall model as well as the Cohen's f^2^ for the individual independent variable); CVR = cerebrovascular reactivity, FA = fractional anisotropy, GMV = grey matter volume, L1 = axial diffusivity, MD = mean diffusivity; p_corr_ = significance level after Benjamini‐Hochberg correction for multiple comparisons, with p_corr_ < 0.05 indicating a significant association after correction. RD = radial diffusivity, WM = white matter, WMH = white matter hyperintensity. 95% CI = 95% Confidence interval.

* Indicates *p* < 0.05. All models included age and sex as covariates. Correction for multiple comparisons was performed within each set of metrics (e.g., all FA measures across the four white matter tracts), thus adjusted *p*‐values reflect this within‐metric correction.

Hippocampal CVR at MRI‐Wave 2 was not significantly associated with longitudinal change in hippocampal volume between MRI‐Wave 1 and 2 (Left: *β* = 1.37, *p* = 0.121; Right: *β* = 1.11, *p* = 0.176, Table 3, Figure 2B).

**TABLE 3:** Summary of statistical associations between CVR (MRI‐Wave 2) and changes in structural measures (MRI‐Wave 2—MRI‐Wave 1).

	Independent variable	Dependent variable	*β*	95% CI	*p*	p_corr_	Cohen's f^2^ Overall model (Independent variable)
GMV	Left hippocampus CVR	Left hippocampus ΔGMV	1.37	[−0.32, 3.05]	0.111	—	—
	Right hippocampus CVR	Right hippocampus ΔGMV	1.11	[−0.50, 2.71]	0.176	—	—
WM microstructure	Δ FA						
	Hippocampal CVR	Corpus callosum	1.22	[0.11, 2.33]	0.032*	0.045*	0.31 (0.03)
		Cingulum bundle	1.31	[0.12, 2.50]	0.031*	0.045*	0.26 (0.03)
		Internal capsule	1.14	[0.09, 2.18]	0.034*	0.045*	0.09 (0.04)
		Fornix	0.22	[−0.89, 1.34]	0.692	0.692	—
	Δ MD						
	Hippocampal CVR	Corpus callosum	−0.64	[−1.57, 0.30]	0.183	0.244	—
		Cingulum bundle	−1.41	[−2.66, −0.15]	0.028*	0.092	0.08 (0.03)
		Internal capsule	−1.20	[−2.39, −0.02]	0.046*	0.092	0.07 (0.03)
		Fornix	−0.20	[−1.19, 0.80]	0.694	0.694	—
	Δ RD						
	Hippocampal CVR	Corpus callosum	−1.08	[−2.13, −0.04]	0.042*	0.056	0.31 (0.03)
		Cingulum bundle	−1.41	[−2.57, −0.26]	0.017*	0.037*	0.21 (0.04)
		Internal capsule	−1.31	[−2.39, −0.22]	0.019*	0.037*	0.11 (0.04)
		Fornix	0.01	[−0.83, 0.84]	0.988	0.988	—
	Δ L1						
	Hippocampal CVR	Corpus callosum	0.05	[−0.94, 1.04]	0.919	0.919	—
		Cingulum bundle	−0.65	[−2.06, 0.76]	0.365	0.487	—
		Internal capsule	−0.88	[−2.06, 0.30]	0.143	0.487	—
		Fornix	−0.94	[−2.76, 0.88]	0.308	0.487	—
WMH	Hippocampal CVR	ΔLogWMH%	−0.24	[−0.81, 0.32]	0.397	—	—

Abbreviations: Δ = standardised change between waves, β = coefficient of the linear regression models, Cohen's f^2^ indicates effect size for significant results, (table displays both Cohen's f^2^ for the overall model as well as the Cohen's f^2^ for the individual independent variable), CVR = cerebrovascular reactivity, FA = fractional anisotropy, GMV = grey matter volume, L1 = axial diffusivity, MD = mean diffusivity, p_corr_ = significance level after Benjamini‐Hochberg correction for multiple comparisons with p_corr_ < 0.05 indicating a significant associations after correction, RD = radial diffusivity, WM = white matter, WMH = white matter hyperintensity. 95% CI = 95% Confidence interval.

* Indicates *p* < 0.05. Correction for multiple comparisons was performed within each set of metrics (e.g., all FA measures across the four white matter tracts), thus adjusted *p*‐values reflect this within‐metric correction. Correction for multiple comparisons was performed within each set of metrics (e.g., all FA measures across the four white matter tracts). Adjusted *p*‐values reflect this within‐metric correction. For each set, four comparisons were corrected.

### 3.3 Associations Between Hippocampal CVR and WM Microstructure

At MRI‐Wave 2, lower hippocampal CVR was significantly associated with poorer WM diffusion metrics in the fornix (lower FA: *β* = 0.09, *p* = 0.045; and higher RD: *β* = −1.9 × 10^−4^, *p* = 0.044), corpus callosum (higher MD: *β* = −1.0 × 10^−4^, *p* = 0.027; and higher RD: *β* = −1.1 × 10^−4^, *p* = 0.035), and internal capsule (higher MD, *β* = −6.5 × 10^−5^, *p* = 0.036; and higher L1: *β* = −1.0 × 10^−4^, *p* = 0.018). These associations did not survive correction for multiple comparisons (p_corr_ > 0.05), and although the overall model demonstrated a small to medium effect size, the effect size for hippocampal CVR as the independent variable was small. Statistical results are summarised in Table 2, Figures 2A and 3A.

Lower hippocampal CVR at MRI‐Wave 2 was also associated with lower preservation of WM microstructural integrity (i.e., larger reductions in WM microstructural integrity) during the preceding decade. These associations were observed in the corpus callosum (significantly larger changes in FA: *β* = 1.22, *p* = 0.032, p_corr_ = 0.045; and RD: β = −1.08, *p* = 0.042, p_corr_ = 0.056), in the cingulum bundle (larger changes in FA: *β* = 1.31, *p* = 0.031, p_corr_ = 0.045; MD: *β* = −1.41, *p* = 0.028, p_corr_ = 0.092; and RD: *β* = −1.41, *p* = 0.017, p_corr_ = 0.037), and in internal capsule (larger changes in FA: *β* = 1.14, *p* = 0.034, p_corr_ = 0.045; MD: *β* = −1.20, *p* = 0.046, p_corr_ = 0.092; and RD: *β* = −1.31, *p* = 0.019, p_corr_ = 0.037). Detailed statistical results are presented in Table 3, Figures 2B and 3B.

### 3.4 Associations Between Hippocampal CVR and WMH

There were no significant associations between hippocampal CVR and total WMH (β = −0.19, *p* = 0.756) or longitudinal changes in WMH (*β* = −0.24, *p* = 0.397, see Tables 2 and 3).

### 3.5 Supplementary Analysis of Whole‐Brain CVR and Brain Structure

Our supplementary analyses revealed no significant associations between whole‐brain CVR and brain structure cross‐sectionally or longitudinally, except between lower whole‐brain CVR and greater longitudinal change in MD (*β* = −1.34, *p* = 0.018, p_corr_ = 0.073) and RD (*β* = −1.10, *p* = 0.037, p_corr_ = 0.147) within the cingulum bundle (Supplementary Table 3).

## 4 Discussion

This study examined the association between hippocampal CVR and brain structural markers of dementia. Our findings revealed that hippocampal CVR was associated with current grey and white matter structure as well as changes in white matter microstructural integrity during the past 9 years, albeit with small effect sizes. Older people with lower hippocampal CVR were likely to have smaller left hippocampal volume, lower WM microstructural integrity across several key white matter tracts, especially in the corpus callosum and internal capsule, and greater WM microstructural integrity decline over the past decade. However, hippocampal CVR was not significantly associated with cerebrovascular lesions as measured by WMH. These findings support the emerging role of hippocampal CVR as a mechanism within the neurodegenerative pathway, and as a marker of brain anatomical and white matter microstructural decline.

Given that the hippocampus is among the first regions to exhibit atrophy in Alzheimer's disease (Pini et al. 2016; Jack et al. 2011; Jack et al. 2000), understanding factors that impact its volume and connectivity could provide insights into early pathological processes. Here, we observed that lower hippocampal CVR was associated with lower hippocampal volume in the left hemisphere, but not in the right. While the exact mechanism underlying this asymmetry remains unclear, the observed associations only in the left hippocampus might be explained by its greater susceptibility to age‐related neurodegeneration. Previous studies suggest functional lateralisation within the hippocampus: the right hippocampus is more involved in spatial memory, while the left is linked to episodic memory, the latter being one of the earliest cognitive functions affected in both ageing and dementia (Ezzati et al. 2016; Tromp et al. 2015). Consistently, studies have shown greater volume reduction in the left hippocampus compared to the right in individuals with MCI and Alzheimer's disease (Shi et al. 2009). It is possible that hippocampal atrophy, which is often a result of neuronal loss (Scheff and Price 2003; Kril et al. 2004), may be exacerbated by impaired CVR, particularly in the left hippocampus, eventually compromising perfusion in this region (Nur et al. 2009). Ji and colleagues noted that the hippocampus has relatively low vascular density, with microvessels spaced further apart (Ji et al. 2021), which makes it more vulnerable to even minor declines in vascular autoregulation and perfusion. This vascular sensitivity may impact both hippocampal volume and neuronal function and contribute to subsequent cognitive decline (Nur et al. 2009; Johnson 2023). Our findings suggest that maintaining hippocampal cerebrovascular reserve could play a role in preserving hippocampal structure in ageing.

We found that lower hippocampal CVR was associated with lower WM microstructural integrity in the corpus callosum, fornix, and internal capsule, along with greater WM microstructural integrity change in the corpus callosum, cingulum bundle, and internal capsule between ~70 and ~80 years of age. While certain associations did not survive multiple comparison correction, their consistent direction and significance before correction suggest potential underlying relationships and are worth discussing. Specifically, we observed associations between higher diffusivity alongside lower FA, indicating a structural loss of myelin in these tracts. The fornix and cingulum bundle are key hippocampal tracts; the fornix connects the hippocampus to other limbic regions essential for memory and emotional regulation (Chen et al. 2015), whereas the cingulum bundle, which links the cingulate gyrus with the hippocampus and other cortical regions, is involved in executive control, emotion, and episodic memory (Bubb et al. 2018). Our results suggest that the microstructural integrity of these output tracts is closely linked to the vascular health of the hippocampus. While our study is not designed to infer causality, one possible interpretation is that impairments in hippocampal vascular reserve could, over time, lead to axonal hypoperfusion and ischemia (Chen et al. 2013; Van Beek et al. 2008), thus disrupting axon‐glia integrity (McQueen et al. 2014). Others have shown that such disruptions are often accompanied by a decrease in the density of oligodendrocytes (the cells responsible for myelination), which further exacerbates myelin loss (McIver et al. 2010; Arai 2020). Interestingly, we also note associations of hippocampal CVR with two distal WM tracts that are not directly connected to the hippocampus but are known to be affected in Alzheimer's disease and MCI: the corpus callosum, which regulates inter‐hemispheric cognitive integration (Barbaresi et al. 2024), and the internal capsule, which is involved in sensorimotor control (Liu et al. 2021). The longitudinal findings in our study highlight the progressive vulnerability of these tracts over time, particularly in the corpus callosum and internal capsule, which exhibited the most pronounced WM microstructural changes. Although these tracts are not anatomically connected to the hippocampus, they may share overlapping vascular territories supplied by branches of the posterior cerebral artery (Johnson 2023; Goldstein et al. 2023; Vitosević et al. 2005). As such, reduced hippocampal CVR can reflect insufficient blood supply that also affects the integrity of these distal tracts, in regions that are metabolically demanding and critical for cognitive function. Previous studies have shown that individuals with higher vascular risk have reduced structural integrity of the corpus callosum and internal capsule (Kennedy and Raz 2009), as well as lower CVR across multiple brain regions, including the hippocampus (Wang et al. 2023). Hence, the associations observed in distal tracts may represent downstream effects of broader vascular insufficiency, partially captured through hippocampal CVR. Importantly, our findings appear to be predominantly localised to hippocampal CVR rather than being globally diffuse, as supplementary analyses revealed no significant associations between whole‐brain CVR and global grey matter atrophy or WM microstructural integrity except in the cingulum bundle. This underscores the critical role of hippocampal vascular reserve in preserving these tracts and, potentially, their associated cognitive functions.

Previous studies have found CVR to be lower in individuals with more pronounced WMH, or within WMH lesions compared to contralateral normal‐appearing white matter in people with leukoaraiosis (Ni et al. 2021; Sam et al. 2016; Marstrand et al. 2002). However, we found no association of hippocampal CVR with total WMH volume. This discrepancy may be due to methodological differences; for instance, one study using pseudo‐continuous arterial spin labelling (pCASL) CVR reported an association between whole‐brain CVR and total WMH volume (Kapoor et al. 2024), whereas our BOLD‐based CVR measures did not show this association. pCASL quantifies cerebral blood flow directly, while BOLD reflects deoxyhaemoglobin‐related signal changes influenced by multiple physiological factors (Liu et al. 2019). It is also likely that the Heart and Brain cohort participants generally had lower WMH burden and less variability in the WMH volumes compared to individuals with more pronounced leukoaraiosis. Given that the role of WMH in dementia primarily depends on lesion severity (Van Der Flier et al. 2005; Frisoni et al. 2007), the lack of association in our study might be explained by the relatively low WMH burden in our cohort. In addition, we assessed total WMH volume rather than regional subtypes (e.g., periventricular or deep WMH), whereas prior studies have shown regionally specific links of WMH subtypes to vascular dysfunction or dementia risk (Low et al. 2022; Vuorinen et al. 2015; Lee et al. 2022). Finally, previous studies have spatially matched CVR measurements to WMH lesion locations (such as assessing CVR within the WMH lesions) (Sleight et al. 2024), whereas we focused on hippocampal CVR.

Several limitations of the study should be acknowledged. First, WHII is a long‐running cohort (~40 years) and therefore susceptible to survival and/or selection bias. The participants are predominantly well‐educated, male, and White British and the findings are hence limited in generalisability. Some significant results did not withstand multiple comparison adjustments, likely due to the sample size and small effect sizes (Cohen's f^2^ < 0.15 (Cohen 2013)). Although the effect sizes in our study were small, they appear comparable to those in other studies with similar sample sizes investigating the associations between CVR and cerebral perfusion (Jefferson et al. 2018). Additionally, since different MRI scanners were used between MRI‐Wave 1 and MRI‐Wave 2, we standardised the structural data separately for each wave to account for inter‐scanner variability. While necessary, this approach limited our ability to capture absolute changes in brain structural health measures for each participant. Instead, it focused on capturing an individual's trend relative to the rest of the cohort, potentially reducing the sensitivity of our longitudinal findings. Finally, our findings should be interpreted with caution, given the limitation that CVR was measured only at follow‐up, whereas changes in brain structure were evaluated retrospectively over the preceding decade. This temporal limitation prevents us from establishing CVR as a predictive marker of these longitudinal changes or inferring directionality and causality in these associations; instead, our results reflect only concurrent associations between CVR and ongoing brain structural decline. Thus, although we observed associations between left hippocampal CVR and GMV, we cannot exclude the possibility that lower CVR may itself be a result of reduced vascular density due to hippocampal atrophy. Nonetheless, previous studies suggest that cerebrovascular dysfunction may precede other biomarkers of Alzheimer's disease. For example, reductions in cerebral blood flow have been shown to emerge earlier than amyloid accumulation and structural atrophy in the progression of Alzheimer's disease (Iturria‐Medina et al. 2016). It has also been suggested that when cerebral blood flow falls below a critical threshold, it may initiate a cascade of reduced neuronal energy, synaptic dysfunction, accumulation of Alzheimer's pathology, and eventually neuronal loss (J. de la Torre 2018; J. C. De La Torre 2012). In line with these mechanistic frameworks, we propose that our follow‐up measurement of CVR likely reflects chronic cerebrovascular health and demonstrates consistent associations with neurodegenerative structural changes in older age. In order to further establish the causal relationships between CVR and brain structural decline, future work should investigate longitudinal assessments of CVR in larger cohorts to verify the retrospective findings from this study. In addition, future studies could examine regional CVR measures beyond the hippocampus to better understand spatial patterns of CVR–white matter microstructure associations. In particular, voxel‐wise analyses in larger samples could help explore associations between regional CVR and adjacent white matter tracts, such as CVR surrounding the corpus callosum or between other white matter tracts with hippocampal CVR.

CVR serves as a marker of brain vascular reserve and is potentially modifiable. Many studies have shown links between CVR and lifestyle risk factors for dementia (Wang et al. 2023), and several preliminary studies have already provided evidence of improvements in CVR, including through hypertension management (Peng et al. 2018), aerobic exercise (Bailey et al. 2013; Guadagni et al. 2020), and inflammation reduction across patient populations (Chiarelli et al. 2022). This highlights the potential for interventions to preserve or even restore vascular reserve, which we suggest may support brain structural health. However, further longitudinal studies are needed to determine whether enhancing hippocampal CVR through such approaches can delay or mitigate neurodegenerative changes.

## 5 Conclusion

In summary, we find that lower hippocampal CVR is associated with dementia‐related biomarkers such as smaller hippocampal volumes and poorer white matter microstructural integrity in key tracts relevant for cognition. The findings suggest that impairments in hippocampal CVR may reflect ongoing hippocampal neurodegeneration and may play an important role in the pathophysiological processes relevant to dementia (Sur et al. 2020; Liu et al. 2024). Since CVR is potentially modifiable, future work could examine whether maintaining hippocampal CVR could preserve brain structure and function, and possibly reduce the risk of cognitive decline.

## Author Contributions

Data collection was conducted by Congxiyu Wang, Graham Reid, Lucy Jobbins, Georgina Hobden, and Sana Suri. CVR data analysis was performed by Congxiyu Wang, with assistance from Graham Reid, Joana Pinto, and Daniel Bulte. FLAIR analysis was carried out by Georgina Hobden. Statistical analysis was conducted by Congxiyu Wang, with assistance from Raihaan Patel. Other imaging analyses were performed by Congxiyu Wang. Congxiyu Wang wrote the manuscript, with all authors reviewing and providing feedback. Klaus P. Ebmeier was responsible for the use of MRI‐Wave 1 data. Sana Suri supervised the Heart and Brain Project and this study, with additional supervision provided by Clare E. Mackay. All authors reviewed and approved the final manuscript.

## Funding

This work was supported by NIHR Oxford Health Biomedical Research Centre, NIHR203316; The HDH Wills 1965 Charitable Trust; Healthy minds from 0‐100 years: Optimising the use of European brain imaging cohorts (Lifebrain) (EU Horizon 2020 Programme Grant agreement ID: 732592); British Heart Foundation, RG/13/2/30098; Wellcome Trust, 203139/A/16/Z, 203139/Z/16/Z, 221854/Z/20/Z; UK Medical Research Council, G1001354/1, MR/K013351/1, R024227, S011676; National Institute on Aging (NIA), National Institutes of Health (NIH), United States (R01 AG056477; RF1 AG062553); University of Oxford, HMD00740.HQ02.01; The Academy of Medical Sciences/the Wellcome Trust/the Government Department of Business, Energy and Industrial Strategy/the British Heart Foundation/Diabetes UK Springboard Award (SBF006/1078); UK Alzheimer's Society, 441.

## Ethics Statement

This study was approved by the Medical Sciences Interdivisional Research Ethics Committee at the University of Oxford (reference number: R57135/RE006).

## Consent

All participants provided written informed consent prior to participation.

## Conflicts of Interest

The authors declare no conflicts of interest.

## Supporting information


**Supplementary Table 1:** MRI acquisition parameters for the two MRI‐Waves (adapted from the protocol paper (Suri et al. 2021)).
**Supplementary Table 2**: Summary of associations between whole brain CVR and structural measures at MRI‐Wave 2.
**Supplementary Table 3**: Summary of associations between whole brain CVR at MRI‐Wave 2 and changes in MRI measures (MRI‐Wave 2—MRI‐Wave 1).
**Supporting Information Figure 1**: End‐tidal CO_2_ trace of a representative participant.
**Supporting Information Figure 2**: Distribution of standardised changes in grey matter volume and white matter metrics.

## Data Availability

The data from MRI‐Wave 1 are available through the Dementias Platform UK (DPUK) portal (https://portal.dementiasplatform.uk/Apply). The data of MRI‐Wave 2 (Heart and Brain Study) will be made accessible via the DPUK portal within 5 years of study completion by 2028. Researchers can access data used in this study through an application to DPUK.
