# Supplementary material for: The Relationship Between Hippocampal Cerebrovascular Reactivity and Brain Structure in Older Age
Source: Hum Brain Mapp. 2026 Feb 18;47(3):e70445. doi: 10.1002/hbm.70445 (PMC12916246; doi:10.1002/hbm.70445)
Supplement: Supplementary file 1 — Supplementary Table 1: MRI acquisition parameters for the two MRI‐Waves (adapted from the protocol paper (Suri et al. 2021)). Supplementary Table 2: Summary of associations between whole brain CVR and structural measures at MRI‐Wave 2. Supplementary Table 3: Summary of associations between whole brain CVR at MRI‐Wave 2 and changes in MRI measures (MRI‐Wave 2—MRI‐Wave 1). Supporting Information Figure 1: End‐tidal CO2 trace of a representative participant. Supporting Information Figure 2: Distribution of standardised changes in grey matter volume and white matter metrics. [file HBM-47-e70445-s001.docx]

**Supplementary Materials**

**Supplementary Table 1: MRI acquisition parameters for the two MRI-Waves (adapted from the protocol paper** ^1^**)**

MRI: Magnetic resonance imaging, BOLD: Blood oxygenation level dependent, TR: Repetition time, TE: Echo time, TI: Inversion time, DTI: Diffusion tensor imaging, A: Anterior, P: Posterior, FLAIR: Fluid-attenuated inversion recovery, MP-RAGE: Magnetisation Prepared - RApid Gradient Echo, MEMPR: Multi-Echo MPRAGE

| **MRI** | **MRI-Wave 1 3T Verio** | **MRI-Wave 2 3T Prisma** |
| --- | --- | --- |
| **Duration** | April 2012–December 2014 | October 2019–May 2023 |
| **Head coil** | 32-channel | 64-channel |
| **Respiratory-calibrated BOLD functional MRI (CVR)** | | |
| Voxel, mm^3^ | -- | 2.4×2.4×2.4 |
| TR/TE, ms | -- | 800/30 |
| Flip angle, ◦ | -- | 52 |
| Field of view, mm | -- | 216 |
| Multi-band acceleration factor *N* volumes | -- | 6 |
| *N* volumes | -- | 450 |
| Acquisition time | -- | 6min 8s |
| **T1** | | |
| Sequence | MEMPR | MPRAGE |
| Voxel, mm^3^ | 1×1×1 | 1×1×1 |
| TR, ms | 2,530 | 1,900 |
| TE, ms | 1.79/3.65/5.51/7.37 | 3.97 |
| TI, ms | 1,380 | 904 |
| Flip angle, ◦ | 7 | 8 |
| Field of view, mm | 256 | 192 |
| Acquisition time | 6min12s | 5 min 31 s |
| **DTI** | | |
| Voxel, mm^3^ | 2×2×2 | 2×2×2 |
| TR/TE, ms | 8,900/91.2 | 8,900/91.2 |
| b-value, s/mm^2^ | 1,500 | 1,500 |
| *N* volumes (A >> P) | 60 + 5*b* = 0 s | 60 + 5*b* = 0 s |
| *N* volumes (P >> A) | 1*b* = 0 | 1*b* = 0 |
| Field of view, mm | 192 | 192 |
| Acquisition time | 9min56s | 10min 5s |
| **FLAIR** | | |
| Voxel, mm^3^ | 0.4×0.4×3.0 | 0.4×0.4×3.0 |
| TR/TE, ms | 9,000/73 | 9,000/73 |
| TI, ms | 2,500 | 2,500 |
| Flip angle, ◦ | 150 | 150 |
| Field of view, mm | 220 | 220 |
| Acquisition time | 4min14s | 4min 14s |

**Supplementary Table 2: Summary of associations between whole brain CVR and structural measures at MRI-Wave 2**

β = coefficient of the linear regression models, CVR = cerebrovascular reactivity, FA = fractional anisotropy, GMV = grey matter volume, L1 = axial diffusivity, MD = mean diffusivity, p_corr_ = significance level after Benjamini-Hochberg correction for multiple comparisons, with p_corr_< 0.05 indicating a significant association after correction. RD = radial diffusivity, WM = white matter, WMH = white matter hyperintensity. 95%CI = 95% Confidence interval. Models included age and sex as covariates. Correction for multiple comparisons was performed within each set of metrics (e.g., all FA measures across the four white matter tracts), thus adjusted p-values reflect this within-metric correction.

|  | **Independent variable** | **Dependent variable** | **β** | **95%CI** | **p** | **p_corr_** |
| --- | --- | --- | --- | --- | --- | --- |
| **GMV** | Whole brain CVR | Whole brain GMV | 13.19 | [-74.43, 100.81] | 0.766 | - |
| **WM microstructure** | **FA** | | | | | |
|  | Whole brain CVR | Whole brain | -0.006 | [-0.05, 0.04] | 0.802 | - |
|  |  | Corpus callosum | -0.01 | [-0.08, 0.05] | 0.632 | .903 |
|  |  | Cingulum bundle | -0.01 | [-0.07, 0.05] | 0.724 | .903 |
|  |  | Internal capsule | -0.003 | [-0.05, 0.05] | 0.903 | .903 |
|  |  | Fornix | 0.03 | [-0.04, 0.11] | 0.423 | .903 |
|  | **MD** | | | | | |
|  | Whole brain CVR | Whole brain | 1.5x10^-5^ | [-4.6x10^-5^, 7.6x10^-5^] | 0.637 | - |
|  |  | Corpus callosum | -8.5x10^-6^ | [-9.1x10^-5^, 7.4x10^-5^] | 0.838 | .912 |
|  |  | Cingulum bundle | 2.1x10^-5^ | [-4.3x10^-5^, 8.4x10^-5^] | 0.522 | .912 |
|  |  | Internal capsule | -3.1x10^-6^ | [-5.7x10^-5^, 5.1x10^-5^] | 0.912 | .912 |
|  |  | Fornix | -3.0x10^-5^ | [-1.7x10^-4^, 1.1x10^-4^] | 0.670 | .912 |
|  | **RD** | | | | | |
|  | Whole brain CVR | Whole brain | 1.4x10^-5^ | [-5.1x10^-5^, 7.9x10^-5^] | 0.675 | - |
|  |  | Corpus callosum | 6.6x10^-6^ | [-8.5x10^-5^, 9.8x10^-5^] | 0.886 | .975 |
|  |  | Cingulum bundle | 1.8x10^-5^ | [-5.3x10^-5^, 8.9x10^-5^] | 0.622 | .975 |
|  |  | Internal capsule | 9.5x10^-7^ | [-5.9x10^-5^, 6.1x10^-5^] | 0.975 | .975 |
|  |  | Fornix | -5.2x10^-5^ | [-2.2x10^-4^, 1.2x10^-4^] | 0.547 | .975 |
|  | **L1** | | | | | |
|  | Whole brain CVR | Whole brain | 1.6x10^-5^ | [-4.3x10^-5^, 7.5x10^-5^] | 0.589 | - |
|  |  | Corpus callosum | -3.9x10^-5^ | [-1.3x10^-4^, 5.6x10^-5^] | 0.418 | .787 |
|  |  | Cingulum bundle | 2.6x10^-5^ | [-6.6x10^-5^, 1.2x10^-4^] | 0.571 | .787 |
|  |  | Internal capsule | -1.1x10^-5^ | [-8.7x10^-5^, 6.5x10^-5^] | 0.774 | .787 |
|  |  | Fornix | 1.5x10^-5^ | [-9.4x10^-5^, 1.2x10^-4^] | 0.787 | .787 |
| **WMH** | Whole brain CVR | LogWMH% | 0.549 | [-0.51, 1.60] | 0.305 | - |

**Supplementary Table 3: Summary of associations between whole brain CVR at MRI-Wave 2 and changes in MRI measures (MRI-Wave 2 - MRI-Wave 1)**.

Δ = standardised change between waves, β = coefficient of the linear regression models, Cohen’s f^2^ indicates effect size for significant results, (table displays both Cohen's f² for the overall model as well as the Cohen's f² for the individual independent variable), CVR = cerebrovascular reactivity, FA = fractional anisotropy, GMV = grey matter volume, L1 = axial diffusivity, MD = mean diffusivity, p_corr_ = significance level after Benjamini-Hochberg correction for multiple comparisons, with p_corr_< 0.05 indicating a significant associations after correction. RD = radial diffusivity, WM = white matter, WMH = white matter hyperintensity. 95% CI = 95% Confidence interval, * indicates p < 0.05. Correction for multiple comparisons was performed within each set of metrics (e.g., all FA measures across the four white matter tracts), thus adjusted p-values reflect this within-metric correction.

|  | **Independent variable** | **Dependent variable** | **β** | **95% CI** | **p** | **p_corr_** | **Cohen's f^2^ Overall model (Independent variable)** |
| --- | --- | --- | --- | --- | --- | --- | --- |
| **GMV** | Whole brain CVR | ΔWhole brain GMV | 0.33 | [-0.62, 1.28] | 0.498 | - | - |
| **WM microstructure** | **ΔFA** | | | | | | |
|  | Whole brain CVR | Whole brain | 0.42 | [-0.51, 1.35] | 0.374 | - | - |
|  |  | Corpus callosum | 0.82 | [-0.18, 1.82] | 0.106 | 0.188 | - |
|  |  | Cingulum bundle | 0.83 | [-0.23, 1.90] | 0.126 | 0.188 | - |
|  |  | Internal capsule | 0.70 | [-0.24, 1.64] | 0.141 | 0.188 | - |
|  |  | Fornix | 0.12 | [-0.88, 1.11] | 0.819 | 0.819 | - |
|  | **ΔMD** | | | | | | |
|  | Whole brain CVR | Whole brain | -0.28 | [-1.03, 0.48] | 0.468 | - | - |
|  |  | Corpus callosum | -0.23 | [-1.07, 0.61] | 0.595 | 0.715 | - |
|  |  | Cingulum bundle | -1.34 | [-2.45, -0.23] | **0.018*** | 0.073 | 0.09(0.04) |
|  |  | Internal capsule | -0.66 | [-1.72, 0.40] | 0.219 | 0.439 | - |
|  |  | Fornix | -0.16 | [-1.05, 0.72] | 0.715 | 0.715 | - |
|  | **ΔRD** | | | | | | |
|  | Whole brain CVR | Whole brain | -0.27 | [-1.05, 0.51] | 0.495 | - | - |
|  |  | Corpus callosum | -0.65 | [-1.59, 0.28] | 0.170 | 0.227 | - |
|  |  | Cingulum bundle | -1.10 | [-2.13, -0.07] | **0.037*** | 0.147 | 0.20(0.03) |
|  |  | Internal capsule | -0.73 | [-1.70, 0.25] | 0.144 | 0.227 | - |
|  |  | Fornix | 0.03 | [-0.71, 0.78] | 0.927 | 0.927 | - |
|  | **ΔL1** | | | | | | |
|  | Whole brain CVR | Whole brain | -0.25 | [-1.10, 0.60] | 0.562 | - | - |
|  |  | Corpus callosum | 0.37 | [-0.51, 1.25] | 0.406 | 0.541 | - |
|  |  | Cingulum bundle | -0.90 | [-2.15, 0.34] | 0.155 | 0.541 | - |
|  |  | Internal capsule | -0.22 | [-1.27, 0.84] | 0.685 | 0.685 | - |
|  |  | Fornix | -0.70 | [-2.32, 0.92] | 0.393 | 0.541 | - |
| **WMH** | Whole brain CVR | ΔLogWMH% | 0.14 | [-0.37, 0.65] | 0.593 | - | - |

**Supplementary Materials Figure 1: End-tidal CO_2_ trace of a representative participant**

*Raw end-tidal CO_2_ (EtCO_2_) trace throughout the hypercapnia challenge. Each green peak corresponds to a single breath, and the orange line represents the detected EtCO_2_ at the end of expiration. The orange trace was resampled at each fMRI repetition time (TR) to align with BOLD data. The breathing paradigm consisted of alternating blocks of medical air (0.04% CO_2_) and hypercapnic gas (5% CO_2_ in air), with 60 seconds of baseline air followed by two 75-second blocks of CO_2_, interleaved with air. For each hypercapnia block, the trace indicates that EtCO_2_ reached a steady state.*

**Supplementary Materials Figure 2: Distribution of standardised changes in grey matter volume and white matter metrics**

The y-axis represents number of participants. X axis represents relative change in (A) hippocampal volume and white matter diffusion metrics within the (B) corpus callosum (C) cingulum (D) internal capsule and (E) fornix between MRI-Wave 1 and MRI-Wave 2. Orange represents potentially relatively greater degeneration in the MRI measures including more negative standardised ΔFA and ΔGMV values relative to others in the cohort, and more positive standardised ΔMD, ΔRD, and ΔL1. Green represents potentially relatively less degeneration in the MRI measures including more positive standardised Δ values for FA and GMV, and more negative standardised Δ values for MD, RD, and L1. CC = Corpus Callosum, FA = fractional anisotropy, GMV = grey matter volume, L1 = axial diffusivity, MD = mean diffusivity, RD = radial diffusivity.

Reference

1 Suri S, Bulte D, Chiesa ST, Ebmeier KP, Jezzard P, Rieger SW *et al.* Study Protocol: The Heart and Brain Study. *Front Physiol* 2021; **12**. doi:10.3389/FPHYS.2021.643725.
